# Differential impacts of wind and waves on albatross flight performance in two ocean basins

**DOI:** 10.1186/s40462-025-00614-w

**Published:** 2025-12-20

**Authors:** Ian J. Maywar, Richard A. Phillips, Rachael A. Orben, Melinda G. Conners, Scott A. Shaffer, Lesley H. Thorne

**Affiliations:** 1 School of Marine and Atmospheric Sciences, Stony Brook University, Stony Brook, NY 11794 USA; 2 British Antarctic Survey, Natural Environment Research Council, Cambridge, CB3 0ET UK; 3 Department of Fisheries, Wildlife, and Conservation Sciences, Oregon State University, Newport, OR 97365 USA; 4 Western EcoSystem Technologies Inc., Cheyenne, WY 82001 USA; 5 Department of Biological Sciences, San Jose State University, San Jose, CA 95192 USA

**Keywords:** Accelerometer, Albatross, Animal movement, Bio-logging, Dynamic soaring, Wave-slope soaring

## Abstract

**Background:**

Albatrosses can commute long distances to feed during the breeding season by avoiding energetically costly flapping flight. Energy from both wind and waves can be used to sustain soaring and reduce flapping flight, yet most studies of albatross flight have focused solely on the influence of wind.

**Methods:**

To examine how wind and waves allow albatrosses to reduce energetic costs by limiting flapping flight, we analyzed how the flap rates of five albatross species (370 individuals) across two ocean basins varied with wind speed and swell height.

**Results:**

For all study species, soaring using both wind and waves resulted in an 89–93% reduction in the number of flaps per hour and thus more energetically efficient flight. We found notable differences in the relative importance of wind and waves for albatrosses breeding in the Southern Ocean and North Pacific. The flap rates of Southern Ocean species, black-browed (*Thalassarche melanophris*), grey-headed (*T. chrysostoma*), and wandering (*Diomedea exulans*) albatrosses, were better explained by variability in windspeed whereas those of North Pacific species, black-footed (*Phoebastria nigripes*) and Laysan (*P. immutabilis*) albatrosses, were better explained by variability in swell height.

**Conclusions:**

Our results suggest that Southern Ocean species relied more on dynamic soaring by exploiting winds whereas North Pacific species relied more on wave-slope soaring using swells. This divergence in behavior is likely the result of differences in the regional winds and swells between the two ocean basins. Although windspeeds experienced by albatrosses in both oceans were similar, North Pacific species experienced greater swell heights, likely allowing them to extract more wind energy from waves than albatrosses in the Southern Ocean. Our research highlights the importance of both wind and waves for albatross movement and the need to better understand environmental impacts on physiological drivers of foraging energetics to assess responses of seabirds to a rapidly changing climate.

**Supplementary information:**

The online version contains supplementary material available at 10.1186/s40462-025-00614-w.

## Background

For animals that fly or swim, the cost and efficiency of movement are profoundly affected by the motions of the surrounding air or water. Therefore, assessing animal responses to fluid flow can provide important context for animal movement strategies and modulation of energy expenditure [1]. The flight of seabirds and thus their behavior, energetics, and life history are heavily influenced by wind patterns [2–5]. Albatrosses have evolved morphological and behavioral adaptations allowing them to exploit wind and wave energy, enabling foraging trips during breeding of hundreds or thousands of kilometers [6–8]. Oceanic wind and wave patterns are increasingly altered by a changing climate [9, 10], so a detailed understanding of how these physical processes influence albatross flight and energetics is critical for predicting future changes in their distribution and life history [11, 12].

Flapping flight relies on mechanical work to overcome gravity and drag and is energetically costly [13–16]. Albatrosses reduce the energetic costs of flying through soaring [17–20], which allows them to achieve some of the lowest flight costs of any seabird by minimizing flapping [8, 21, 22]. In general, soaring is a behavior where a bird extracts energy from atmospheric movements to fuel and sustain gliding, the conversion of gravitational potential energy into kinetic energy without the need for mechanical work [17]. Dynamic soaring takes advantage of wind shear, the increase of windspeed between horizontal layers of wind due to friction at the sea surface. When dynamic soaring, birds bank into the wind to climb the wind shear gradient and gain elevation, turn away from the wind to glide and cover horizontal distance while losing altitude, and then bank back into the wind to repeat the cycle [19, 23]. The dynamic soaring cycle can be supplemented with infrequent flapping [24]. In addition to dynamic soaring, traveling waves can cause air to move upwards, and albatrosses can utilize wave-slope soaring to take advantage of these wave-fueled updrafts. When doing so, they bank into the direction of the wave to gain elevation from updrafts, turn to glide parallel to wave crests where they are supported by additional updrafts, then bank back into the direction of the wave to repeat the cycle [19, 25]. Dynamic soaring requires high windspeeds to create sufficient wind shear whereas the wave-fueled updrafts necessary for wave-slope soaring require swells of sufficient height, and the latter can be used even in the absence of winds [18, 25, 26]. Albatrosses use flapping flight or sit on the water when wind or waves are insufficient for soaring [17, 19, 27, 28], though extremely strong winds may also present energetic and foraging efficiency challenges [29].

Dynamic soaring cycles relative to wind [17, 18, 30, 31] and wave-slope soaring cycles relative to waves [25] have been mathematically modeled, but with the miniaturization of biologging devices over the last two decades, we can now capture seabird movements *in-situ* at a high resolution [32–34]. Previous studies have used high-resolution GPS data to support the modelling of soaring relative to wind [23, 35–37], and others have additionally used tri-axial inertial measurement units (i.e. accelerometers, magnetometers) to understand turning, angles, flap rates, dynamic body acceleration, body power, and other movement characteristics relative to wind [24, 38, 39]. However, empirical studies of wave-slope soaring and its comparison with dynamic soaring are lacking. The analysis of wave-slope soaring has yet to be conducted *in-situ* using biologging sensors, as previous research has focused on the quantitative modeling of this cyclical behavior [19, 25].

Albatross inhabit regions characterized by persistent, strong winds; 18 of the 22 total albatross species (all *Diomedea, Thalassarche,* and *Phoebetria* species) breed in the Southern Ocean and exploit areas with some of the greatest windspeeds in the world [40]. All four *Phoebastria* species breed in the North Pacific Ocean, where average annual windspeeds are generally lower [40]. Although the importance of wind for albatross movement and habitat use is widely recognized [4, 12, 20], and the impact of ocean-specific environmental differences on albatross behavior and morphology are well studied [6, 40], differences in flight behavior and energetics relative to wind have not been compared across species breeding in different ocean basins. Additionally, tracking studies of albatross flight behavior relative to wind have primarily focused on wandering albatrosses [20, 23, 24, 41], while wind effects on the flight behavior of other albatross species have received less attention. Furthermore, studies of wave usage for albatross species, whether within or across ocean basins, are limited (e.g. [40]). Generally, albatross wings have evolved to facilitate soaring flight at a low cost, with a high aspect ratio (square of wingspan to wing area) and relatively low wing loading (weight per unit wing area) in comparison to aspect ratio, which provide high lift relative to drag [17]. However, the species can differ considerably in their body size and wing morphology, which affects their flight performance and behavior in different wind and wave conditions [4, 18, 40].

Windspeed and wave height are key parameters influencing the energy that albatrosses can gain from their environment [17–19]. Faster winds result in greater windshear, and therefore greater energy available for an albatross banking into the direction of the wind during dynamic soaring. Dynamic soaring is most efficient in crosswinds (sidewinds) or tailwinds, resulting in a preference for these conditions [20, 36, 38, 42]. Upwind flight is less efficient and is generally avoided [40, 43–45]. In wave-slope soaring, higher waves generally result in faster wave-fueled updrafts and more energy available to an albatross alternating between flight that is parallel and perpendicular to the wave direction [19]. Here we assess how wind and waves influence flight in five species of albatrosses breeding in the North Pacific and Southern Ocean. We use flapping rate, a proxy of flight costs in albatrosses [46], to make inferences about how wind and waves influence albatross energetics. To put our results in perspective, we compare the regional availability of wind and waves in the two ocean basins and the conditions experienced by each of our five study species.

## Methods

### Study species

Biologging devices (tags) were deployed on three Southern Ocean albatross species, black-browed (*Thalassarche melanophris*), grey-headed (*T. chrysostoma*), and wandering albatross (*Diomedea exulans*), and two North Pacific albatross species, black-footed (*Phoebastria nigripes*), and Laysan (*P. immutabilis*) albatross. Black-browed, grey-headed, black-footed, and Laysan albatrosses are similar in size, mass, and wing loading (weight per unit wing area) though with some differences [40, 47]. Wandering albatrosses have a body mass that is 2–3 times higher and have the greatest body size and wing loading [48]. The four smaller-bodied species breed in broadly the same months, corresponding to the Southern Hemisphere summer and Northern Hemisphere winter. In the Northwest Hawaiian Islands, black-footed and Laysan albatrosses incubate a single egg from late November until late January, brood-guard lasts for three weeks, and then the chick is provisioned until fledging in June or July [49]. At South Georgia, black-browed and grey-headed albatrosses incubate eggs from late September to December or January, brood-guard also lasts about three weeks, and then the chick is provisioned until fledging occurs in late April to early June [50]. Wandering albatrosses at South Georgia have a much longer breeding cycle; incubation is from mid-December to mid-March, brood-guard lasts about a month, and the chick is fledged in the following November or December [51].

### Study sites

**Fig. 1:**
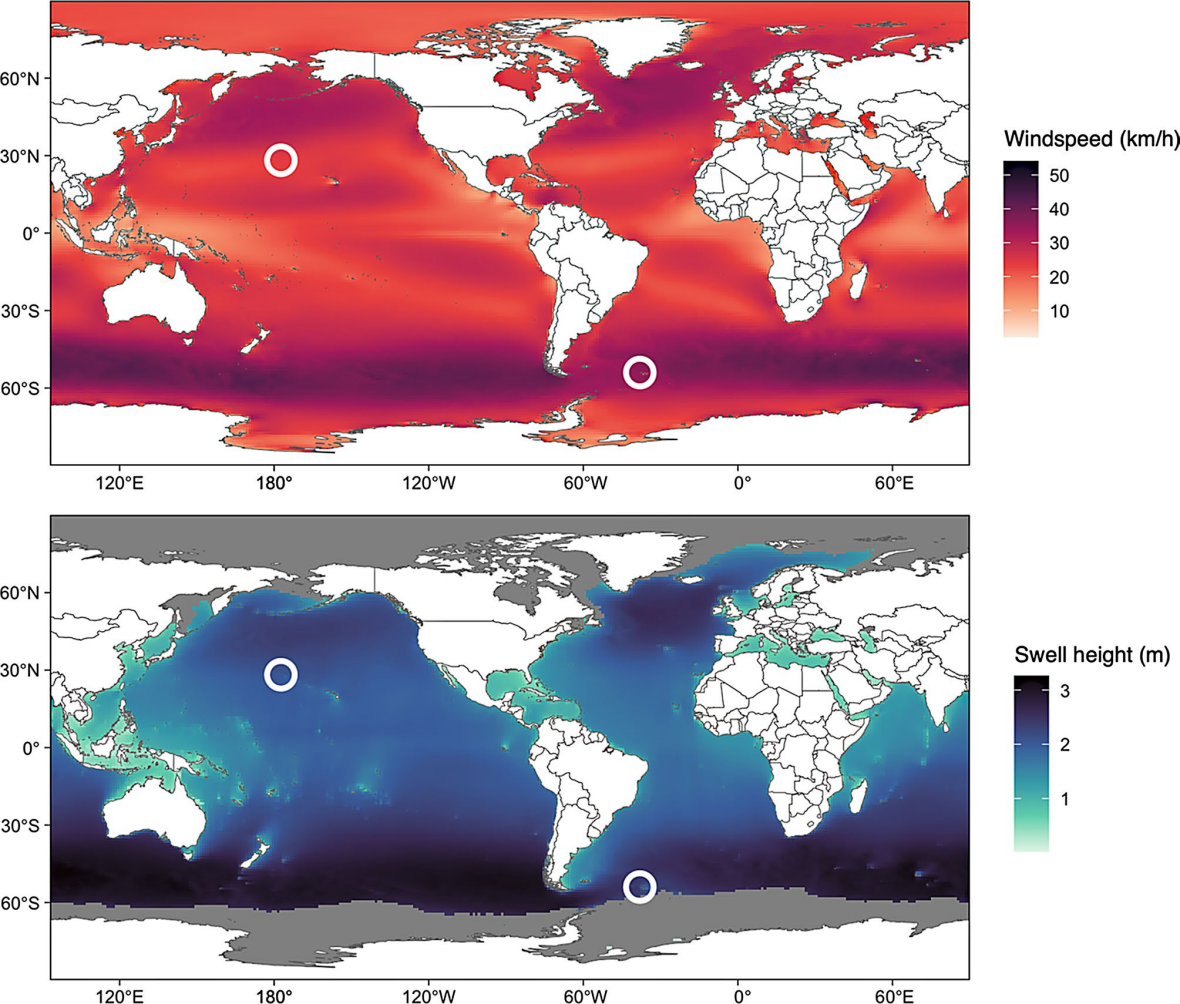
Average annual windspeeds and swell heights during the years of the study (2018–2023). The white circles represent the study sites (Midway Atoll in the Northern Hemisphere, bird Island in the Southern Hemisphere = bird Island). Grey colors represent areas with no wave data due to the presence of sea ice

Fieldwork was conducted at Bird Island, South Georgia (38.03° W, 54.00° S) and Midway Atoll National Wildlife Refuge (177.37° W, 28.21° N) in the Southern Ocean and North Pacific Ocean, respectively (Fig. 1). Approximately 8264 breeding pairs of black-browed albatrosses, 5120 breeding pairs of grey-headed albatrosses, and 859 breeding pairs of wandering albatrosses breed at Bird Island, making up 1.2%, 5.2%, and 10.3% of their global population, respectively [52]. On Midway Atoll, there are approximately 22,000 breeding pairs of black-footed albatrosses and 450,000 breeding pairs of Laysan albatrosses, representing 33.9% and 67.6% of their global population size, respectively [52]. Winds at Midway Atoll show greater seasonal variability, with stronger mid-latitude westerlies occurring in the Northern Hemisphere winter [4]. South Georgia generally experiences mid-latitude westerly winds. Foraging areas used by albatrosses from both breeding sites are generally characterized by large swell heights (Fig. 1). Analyses were performed to identify and compare wind and wave magnitudes experienced by foraging birds at Bird Island and Midway Atoll across time; see Methods: *Wind and wave conditions of foraging areas used during the breeding season* for more detail.

### Tag deployments

Global Positioning System (GPS) and accelerometer tags were deployed on 370 foraging albatrosses: 319 across black-browed, grey-headed, and wandering albatrosses at Bird Island during the 2019/20, 2020/21, and 2021/22 breeding periods, and 51 across black-footed and Laysan albatrosses at Midway Atoll during the 2018/19, 2021/22, and 2022/23 breeding periods (Table 1).

**Table 1:** Summary of tag deployments across field seasons

Species	Site	Field season	Deployments	Deployment duration (hrs)	Flight data duration (hrs)	Flight data duration of complete trips (hours)
Black-browed	Bird Island	2019/20	44	5626	3915	3734
		2020/21	37	4258	3177	4258
		2021/22	53	9018	6705	9018
		**Total**	**134**	**18902**	**13797**	**17010**
Grey-headed	Bird Island	2019/20	43	5449	3776	3082
		2020/21	42	4554	3446	4554
		2021/22	56	9438	6755	9438
		**Total**	**141**	**19441**	**13977**	**17074**
Wandering	Bird Island	2021/22 **(Total)**	**44**	**7680**	**4014**	**6053**
Black-footed	Midway	2018/19	10	486	433	486
	Midway	2022/23	8	1943	1078	1823
		**Total**	**18**	**2429**	**1511**	**2309**
Laysan	Midway	2018/19	15	1373	1072	1316
	Midway	2021/22	9	2602	1747	2515
	Midway	2022/23	9	2577	1953	2577
		**Total**	**33**	**6552**	**4772**	**6408**

Most deployments consisted of separate GPS and accelerometers, and a smaller number of deployments were of multi-sensor devices which recorded both GPS and accelerometer data. Co-deployments consisted of a 22 g CatLog GPS tag (Perthold Engineering, USA) and either a 7.5 g A×y5 or AxyAir tri-axial accelerometer, or a 31.5 g AGM tri-axial accelerometer equipped with a magnetometer and gyroscope (Technosmart, Italy). Multi-sensor deployments used either a 14 g Technosmart-manufactured AxyTrek tag, which included both a tri-axial accelerometer and a GPS, or a custom-built, waterproof, 42 g Neurologger 2A tag (Evolocus, USA), which integrates a miniaturized electrocardiogram, magnetometer, and accelerometer, along with a CatLog GPS [32]. Global Location Sensor (GLS)-immersion tags manufactured by Migrate Technology (UK), with a mass ≤3.3 g which included an immersion (wet-dry) sensor, were deployed on the tarsus of all albatrosses tagged at Bird Island, but not at Midway Atoll. Tape (Tesa 4651 tape, Germany) was used to attach the tags to the central dorsal contour feathers of the albatrosses. The total mass of the devices and tape were < 3% body mass, the recommended threshold for large flying seabirds [53]. GPS data were recorded at 1, 2, 5, or 10-minute intervals and interpolated to 10 minutes. Accelerometer data were recorded at 25 Hz (Technosmart tags) or 75 Hz (Neurologger 2A tags) and downsampled to 25 Hz. The immersion sampling interval of the GLS tags was 3 seconds, where wet-dry states ≥ 2 samples (≥6 seconds) were recorded. Using Animal Tag Tools (http://www.animaltags.org) in MATLAB, tri-axial accelerometer data were rotated horizontally to the animal frame to account for small tag placement errors when the tag was taped in place.

### Characterizing flapping behavior

Wing flaps are a good correlate of energy expenditure in albatrosses [46, 54], and can be seen as spikes in the heave (z) axis of accelerometer data [54–57]. To count individual flaps, we followed Schoombie’s et al. [24] framework of detecting flaps, filtering the z-axis accelerometer signal using LULU operators [58] and detecting peaks above a certain threshold. We incrementally altered the filter and threshold to maximize the ability to detect flaps without capturing noise (additional details provided in Supplemental Information).

### Identifying flight behavior

We used Hidden Markov Models (HMMs) on GPS data to isolate portions of albatross tracks where birds were in flight. Using the moveHMM package [59] in R, step length and turn angle were calculated from GPS data interpolated to 10 minutes to match the temporal resolution of the coarsest-scale GPS data. For each species, these metrics were compiled across all individuals from all field seasons to create a 3-state HMM in moveHMM, representing resting on water, foraging, and commuting states; the latter two states were grouped for our analyses of flap rates. Each GPS datapoint was assigned the state as predicted by the HMM. A subset of co-deployed GLS immersion data from Bird Island were analyzed to validate HMM performance in distinguishing flight in-air versus resting on-water. We classified any 10-minute interval with wet periods in the GLS data as on-water. Comparing on-water states as indicated by the HMM and the GLS, we found that 98.6% of the HMM-classified on-water periods aligned with GLS-classified on-water periods, confirming that the HMM state classification was highly accurate in detecting on-water periods. As immersion tags were only deployed on albatrosses at Bird Island, analyses of flight behavior were based on the HMM state.

### Wind and wave data

Hourly wind and wave data were obtained from the European Centre for Medium-Range Weather Forecasts Reanalysis v5 (ERA5) [60], which has a spatial resolution of 0.25º by 0.25º for wind data and 0.5º by 0.5º for wave data. The 10-meter u and v-components of wind (m/s) were used to calculate windspeed and wind direction at 10 meters above sea-level. Wave parameters included in models were the mean direction (º) and significant height (m) of total swell, the average height of the greatest third of surface sea waves associated with swell [60]. For our flight analyses we focused on the total swell rather than wind waves or sea surface waves, the combination of swell and sea surface waves. Wind waves, and therefore sea surface waves, are created by local winds and are therefore highly correlated with windspeed (Fig. S2), making it difficult to assess impacts of wind vs. waves. Swell height is generated by distant weather systems rather than local winds and is thus not correlated with windspeed (Fig. S2). Since the strength of updrafts is relevant to wave slope soaring and is correlated with wave height, we therefore examined swell height when assessing the impacts of waves on albatross flight behavior.

### Wind and wave conditions of foraging areas used during the breeding season

To provide context for the timing of breeding relative to wind and wave variability we examined seasonal changes in wind and waves within kernel density estimates (KDEs) constructed using foraging tracks from the breeding season. We then examined how the wind and wave conditions within these fixed foraging areas varied across each month of the year. During non-breeding months the study species forage in more distant habitats outside the bounds of the breeding season foraging KDEs [61, 62]. Here we are not assessing how wind and wave conditions experienced by foraging albatrosses vary by month but are rather analyzing how wind availability in proximity to the colony varied by month to determine if birds might be breeding during a period of minimal, maximal or typical wind and wave conditions. Using the adehabitatHR package in R [63], we pooled all complete foraging tracks across all field seasons to calculate the 95^th^ percentile KDE for each species (Fig. S3). Monthly averages of 10-meter windspeed (0.5º by 0.5º spatial resolution) and significant height of total swell (1º by 1º spatial resolution) were downloaded from the ERA5 reanalysis for the study years. We calculated the mean windspeed and swell height for the areas within the KDEs and calculated the multi-year averages for each year of the month.

### Wind and wave conditions along albatross foraging tracks

Wind and wave conditions were extracted along albatross foraging tracks to examine the environmental conditions experienced throughout foraging trips. Since the temporal scale of the wind and wave data extracted from ERA5 was one hour, location and behavioral data were summarized hourly to reflect this resolution. If a bird was predicted by the HMM to have spent time resting on the water during a given hour, this hour was removed to limit analyses to only periods of flight. Flaps, detected from the accelerometer data, were summed for each hour (flaps/hour), and synced with wind and swell data extracted at a birds’ hourly interpolated position along the track. Wind direction was used to calculate the wind direction relative to the bird’s direction of travel, which was assessed using the bird’s GPS track. The resulting “bird-wind angle” (BWA) was assessed on a 0–180º scale such that 0º represents a direct tailwind (as wind direction describes the direction of origin of the wind) and 180º represents a direct headwind. Similarly, the direction of the total swell relative to the bird’s GPS track (BSA: bird-swell angle) was calculated on a 0–180º scale such that 0º is a bird traveling directly with the swell and 180º is a bird traveling directly against the swell.

### Models of flap rates relative to wind and waves

Given the considerable morphological differences across our study species and the impact of morphology on energetic expenditure relative to wind and waves [4, 17, 40], we created separate models of flap rates for each species. We built generalized additive models (GAMs) using the *mgcv* package in R [64] to predict flap rate using windspeed, bird-wind angle, swell height, and bird-swell angle as environmental predictors for each species. To best understand the effects of wind and waves on albatross flap rates, we used a forward selection approach, whereby we began with the simplest models with either windspeed or swell height as the only environmental predictor, then increased the complexity of our models with additional predictors, and identified the best model as the one with the lowest Akaike information criterion (AIC [65–67]). To assess the individual explanatory power of windspeed and swell height on flap rate, we compared the R^2^ value of our models which included only wind and only swell as predictors, respectively.

Our GAMs (Table S1) predicted flap rate using windspeed alone (Model I), swell height alone (Model II), windspeed and bird-wind angle (Model III), swell height and bird-swell angle (Model IV), and both windspeed and swell height together (Model V). We used a full tensor product to capture the interactions between variables for Model III, IV, and V (Table S1). We did not build a model using windspeed and bird-wind angle and its interaction with swell height and bird-swell angle because a model with this level of complexity would be too difficult to interpret. A null model (Model 0) which did not include an environmental predictor, but still included a random effect smooth for individuals, was created for comparison to other models. See Supplemental Information for additional details on modeling parameters.

We trimmed the outputs of Model III, IV, and V, to reflect the 99^th^ percentile of data experienced by each species using kernel density estimates (KDEs). This ensures that environmentally unlikely or impossible values are not interpreted when analyzing these models. The outputs for these models were plotted using colored contours created by Jenks’ natural breaks for visualization purposes [68].

We also plotted the outputs of Model V using line graphs of flap rate versus windspeed or swell height while holding the other explanatory variable constant at the mean value experienced by each species during flight. These plots aim to visualize the individual effect of windspeed and swell height on albatross flapping rates.

### Reduction in flap rate associated with wind and waves

Model V captures changes in flap rates in response to both wind and waves. When visualizing the predicted flap rate from Model V, we constrained the 2D variable space of wind and swell magnitude using 99^th^ percentile KDEs to run Model V for wind and swell conditions that are likely to be experienced by each species. We quantified the reduction in flapping across this simulated 2D space of windspeed and swell height to infer how dynamic and wave-slope soaring in association with wind and waves could influence energy savings in albatross. We calculated the percent reduction in flap rate from the maximum (calculated using the 95^th^ percent quantile) to the minimum (5^th^ percent quantile) of predictions by Model V in the simulated variable space (i.e., [[maximum predicted flap rate – minimum predicted flap rate]/maximum predicted flap rate]).

### Impact of sample size on model results

Our sample size for modeling flap responses of black-footed albatrosses (*N* = 18 individuals) was considerably lower than for any of the other study species (Table 1). To assess whether sample size influenced the flap responses to wind or waves for this species, we ran 100 simulations to create models (Model V) for each species which used 18 individuals and examined variability in the model responses across these simulations. For each simulation, individuals from across all field seasons were randomly selected for each species without replacement. Given that foraging trips during brood-guard are typically shorter than those during incubation, we randomly selected for individuals in each species such that the ratio of brood-guard to incubation trips of black-footed albatrosses (12:6) was preserved.

### Wind and wave conditions experienced by foraging albatrosses

We evaluated windspeed and swell height experienced along the foraging tracks of albatrosses. We removed incomplete foraging trips, in which the GPS stopped recording data before the albatross returned to the colony, to prevent bias introduced by different environmental conditions experienced by albatrosses leaving versus returning to the colony (Table 1). We examined the proportion of time each albatross spent traveling in head-, cross-, and tailwinds (categorized BWA) and against, across, and with the swell (categorized BSA). We also examined the proportion of time albatrosses spent in winds and swell of different intensities, and categorized windspeed and total swell as low, medium, and high, using the same categorizations for all species to facilitate comparisons across species. To establish thresholds, we randomly selected 34 individuals with complete foraging trips (Table S2) from each ocean basin such that there were an equal number of individuals from each species within both ocean basin groups. This was done to ensure that wind and wave magnitudes were not biased by sample size. From this subset of data, the 1/3 and 2/3 quantiles of the total distribution of windspeed and swell height were taken to create breaks for the wind and wave magnitude categorizations.

## Results

### Models of flap rates relative to wind and waves

Our results suggest that windspeed predicted flap rates better than swell height for Southern Ocean albatross species, while for North Pacific species, swell height was a better predictor of flap rate than windspeed. For the three Southern Ocean species, models predicting flap rates from winds outperformed the equivalent models assessing only effects of swell height (i.e., Model I outperformed Model II, and Model III outperformed Model IV), indicating that winds better predicted flap rate than waves (Table 2). In contrast, for the North Pacific species, models predicting flap rates from swell height generally outperformed the equivalent models assessing only effects of wind (i.e., Model III outperformed Model I for both species and Model IV outperformed Model II for black-footed albatrosses while models IV and II performed similarly for Laysan albatrosses), as swell height was generally a more effective predictor of flap rate than windspeed (Table 2). However, across all five study species, the model incorporating effects of both wind and waves (Model V) performed the best (Table 2). The null model (Model 0) performed the worst for all five study species, according to corrected AIC, R^2^, and deviance explained. Further details on the outputs of Models I and II are provided in the Supplemental Information. Models III and IV reveal that wind and wave magnitude had a larger effect on flap rate than relative angle (see Supplemental Information for more detail).

**Table 2:** Model performance for models used to assess the impacts of wind and waves on albatross flapping rates

Species	Model	Environmental term	AICc	dAICc	df	weight	R^2^	DE
Black-browed	0	Null	197436.24	1652.39	124.96	0.00	0.13	0.13
	I	Windspeed	196419.32	635.46	125.33	0.00	0.18	0.18
	II	Swell height	197028.16	1244.31	125.21	0.00	0.16	0.15
	III	Windspeed, BWA	196308.15	524.30	130.05	0.00	0.19	0.19
	IV	Swell height, BSA	196764.18	980.33	130.81	0.00	0.17	0.16
	**V**	**Windspeed, Swell height**	**195783.85**	**0.00**	**129.74**	**1.00**	**0.22**	**0.21**
Grey-headed	0	Null	195469.06	1598.46	134.98	0.00	0.19	0.20
	I	Windspeed	194463.50	592.91	136.45	0.00	0.23	0.24
	II	Swell height	195051.83	1181.24	135.76	0.00	0.21	0.22
	III	Windspeed, BWA	194300.66	430.07	142.26	0.00	0.23	0.25
	IV	Swell height, BSA	194831.72	961.13	141.44	0.00	0.22	0.23
	**V**	**Windspeed, Swell height**	**193870.59**	**0.00**	**140.71**	**1.00**	**0.22**	**0.27**
Wandering	0	Null	56658.72	588.18	42.40	0.00	0.10	0.12
	I	Windspeed	56291.99	221.45	42.93	0.00	0.18	0.18
	II	Swell height	56426.52	355.98	44.30	0.00	0.13	0.16
	III	Windspeed, BWA	56221.35	150.81	49.51	0.00	0.19	0.20
	IV	Swell height, BSA	56319.01	248.47	50.07	0.00	0.16	0.18
	**V**	**Windspeed, Swell height**	**56070.54**	**0.00**	**48.22**	**1.00**	**0.19**	**0.22**
Black-footed	0	Null	20310.47	197.80	17.97	0.00	0.13	0.18
	I	Windspeed	20287.85	175.18	20.13	0.00	0.15	0.19
	II	Swell height	20230.68	118.01	19.88	0.00	0.21	0.22
	III	Windspeed, BWA	20246.05	133.38	26.42	0.00	0.18	0.22
	IV	Swell height, BSA	20205.97	93.30	25.52	0.00	0.21	0.23
	**V**	**Windspeed, Swell height**	**20112.67**	**0.00**	**25.67**	**1.00**	**0.39**	**0.27**
Laysan	0	Null	61837.12	739.59	33.26	0.00	0.15	0.23
	I	Windspeed	61483.33	385.80	35.23	0.00	0.20	0.28
	II	Swell height	61494.60	397.07	35.09	0.00	0.23	0.28
	III	Windspeed, BWA	61418.92	321.39	41.22	0.00	0.23	0.29
	IV	Swell height, BSA	61489.85	392.32	39.96	0.00	0.23	0.28
	**V**	**Windspeed, Swell height**	**61097.53**	**0.00**	**40.88**	**1.00**	**0.25**	**0.33**

**Fig. 2:**
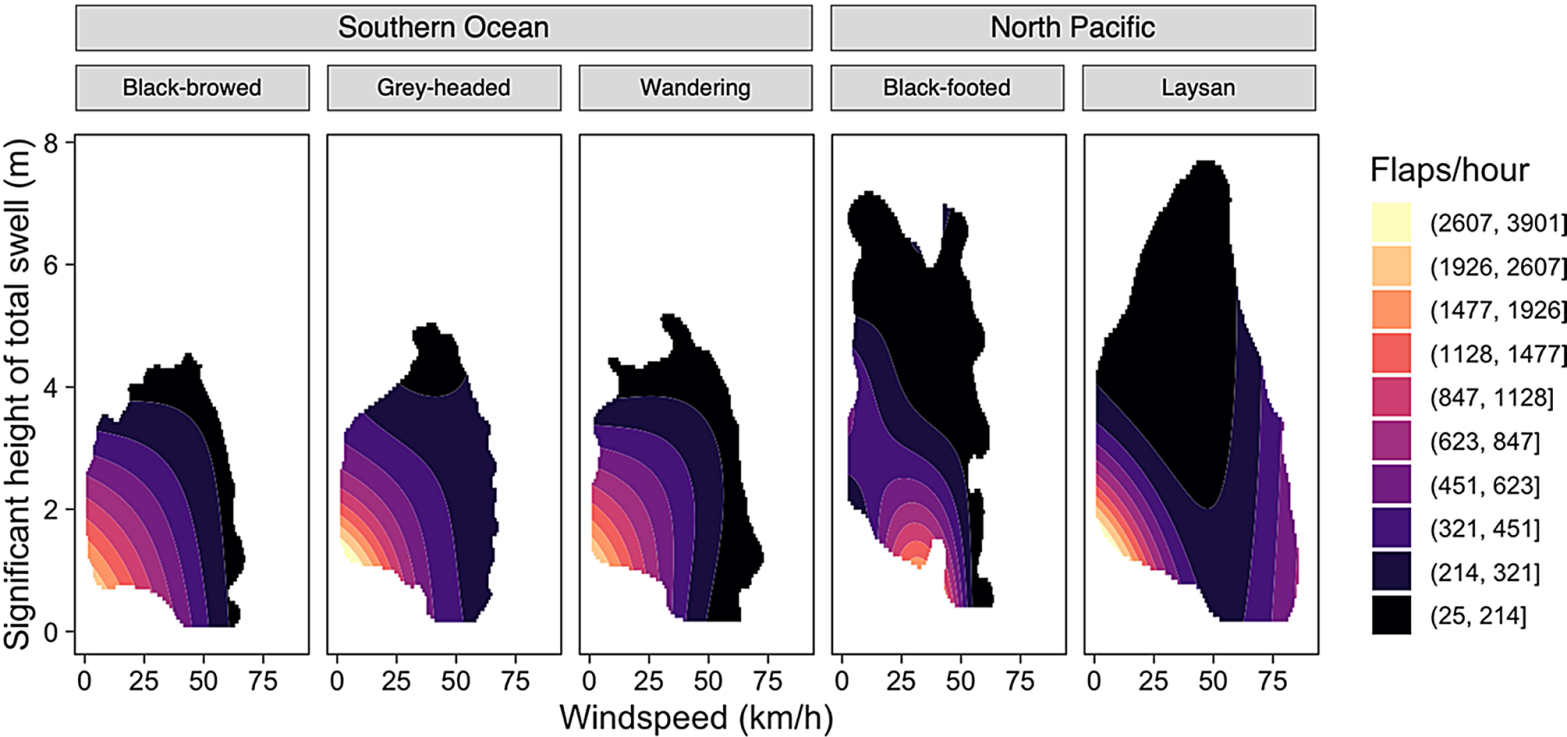
GAMs predicting flap rate using windspeed and swell height (Model V). The outputs of the GAMs were plotted using colored contours created using Jenks’ natural breaks. gam outputs were confined to the 99% kernel density estimate of the predictor variable space to avoid interpreting the output of environmental conditions that are impossible or unlikely

**Fig. 3:**
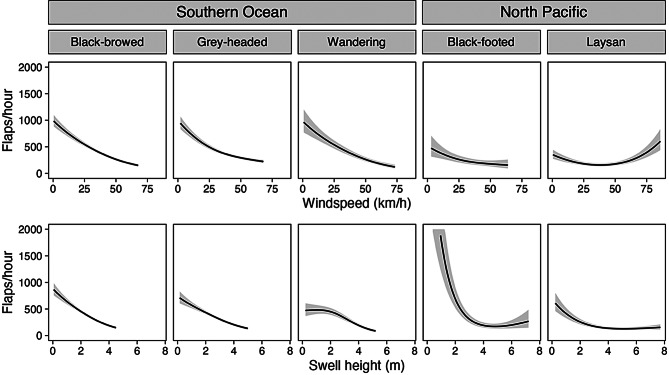
Model V predictions with one environmental variable held constant at the mean experienced value. All plots display the gam predictions and 95% confidence intervals of Model V, the best performing model which use full tensor products of windspeed and swell height. The top row displays the outputs for each species where swell height is held constant at the mean swell height experienced. The bottom row displays the outputs of the models when windspeed is held at the mean windspeed experienced by each species

When the effects of windspeed and swell height on flap rates were examined together (Model V), Southern Ocean albatross species showed convergent responses to wind and waves (Fig. 2). Black-browed, grey-headed, and wandering albatrosses all showed their highest flap rates at low windspeeds and low swell heights. The flap rates for the Southern Ocean species declined with both increasing windspeed and increasing swell heights, generally declining more rapidly with windspeed (Fig. 2). Visualizing the individual effects of windspeed and swell height showed similar responses among Southern Ocean albatross species, with flap rate decreasing with increasing windspeed and swell height, respectively (Fig. 3). Responses to both wind and waves differed in North Pacific species. For Laysan albatrosses, flap rates were elevated at low windspeeds and low swell heights and declined with both increasing swell height but increased at the highest windspeeds (Figs. 2, 3). For black-footed albatrosses, the lowest flap rates were observed at low swell heights and intermediate windspeeds (Fig. 2). When examining the individual effects of windspeed and swell height for black-footed albatross, flap rates decreased markedly with increasing swell heights and decreased minimally with increasing windspeed (Fig. 3).

### Reduction in flapping rate associated with wind and waves

Flap rates were reduced by 89–93% in the five study species when using wind and waves to soar (Table 3). Black-footed albatrosses showed the greatest reduction in flap rate with increases in wind and waves, with a 93.41% reduction from a maximum of 1043 to a minimum of 68.71 flaps per hour (Table 3). Black-browed albatrosses showed the smallest reduction in flap rate, with an 89.19% reduction from a maximum of 1549 to a minimum of 167.5 flaps per hour (Table 3).

**Table 3:** Reduction in flapping rate

Species	Black-browed	Grey-headed	Wandering	Black-footed	Laysan
Maximum flap rate	1549	1952	1512	1043	1429
Minimum flap rate	167.5	160.0	116.4	68.71	129.9
Reduction in flapping (%)	89.19	91.80	92.30	93.41	90.91

### Impact of sample size on model results

The downsampled responses to wind and wave inputs, produced using a reduced sample size (18 tracks, the sample size available for black-footed albatrosses) for all species, were similar to those produced using the entire dataset (Fig. S6). This suggests that the observed responses to wind and waves for black-footed albatrosses is unlikely to be due to the smaller sample size available for this species

### Wind and wave conditions experienced by foraging albatrosses

**Fig. 4:**
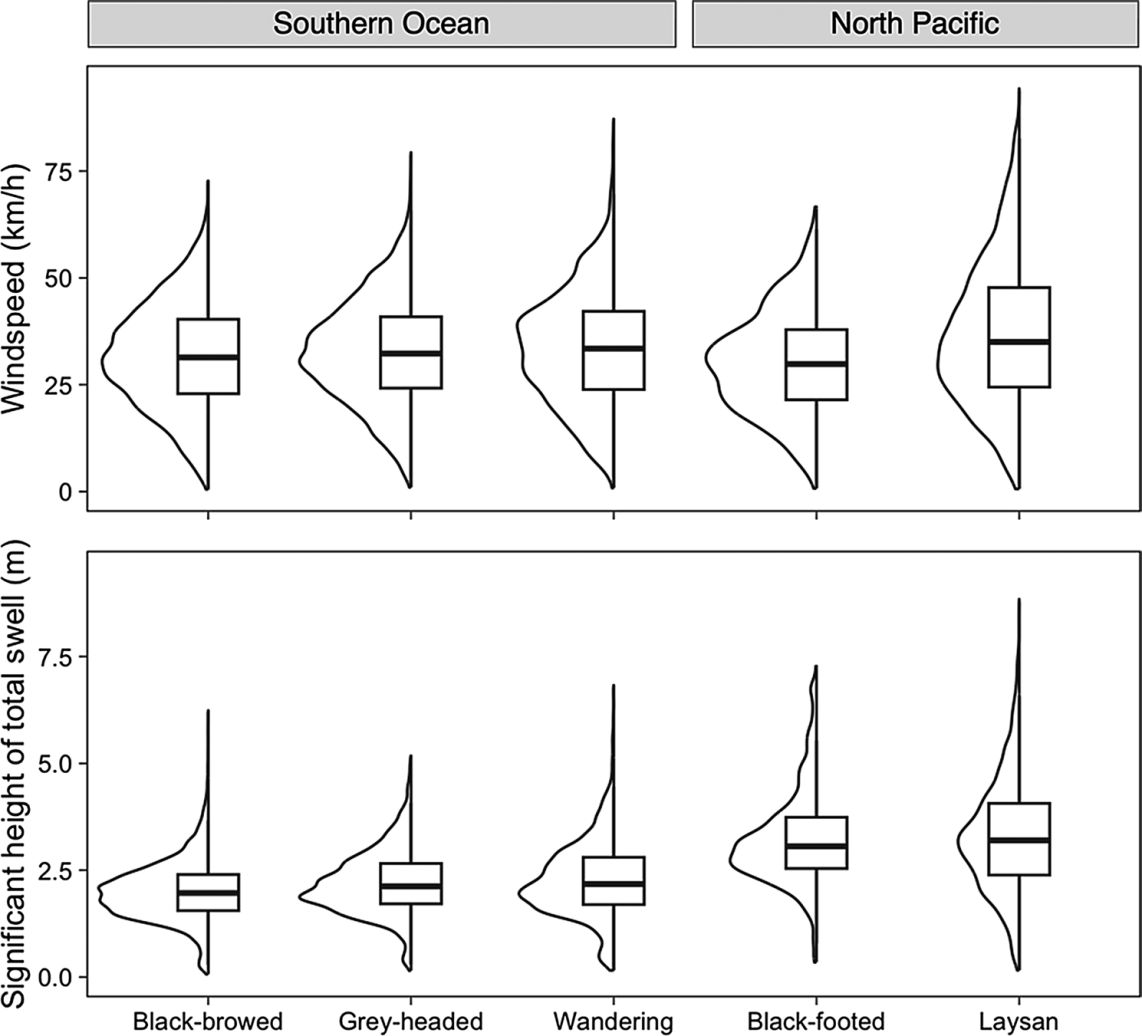
Violin plots overlayed with boxplots of windspeeds (km/h) and significant height of total swells (**m**) experienced along the tracks of foraging albatross across all field seasons

All five albatross species experienced similar windspeeds across their foraging tracks, whereas the species in the North Pacific Ocean experienced greater swell heights than those in the Southern Ocean (Fig. 4). The threshold between low and medium windspeeds was calculated to be 27.1 km/h, while the threshold between medium and high windspeeds was 39.4 km/h. The thresholds for swell heights were 2.18 m and 3.11 m, respectively. The Southern Ocean species displayed no major trends in time spent flying in low, medium, and high windspeeds, whereas in the North Pacific, black-footed albatrosses spent less time in high windspeeds, and Laysan albatrosses spent more time in high windspeeds (Fig. S7). The Southern Ocean study species spent little time flying with high swell heights and generally spent the majority of their time flying with low swell heights. The North Pacific albatrosses flew through mostly high swells and rarely low swells (Fig. S7). When grouped into categories of wind direction (see Methods), all five species largely avoided headwinds and preferred crosswinds, but there was no strong preference for, nor avoidance of particular swell angles (Fig. S8). During the breeding season, the species breeding at Midway Atoll typically travel north of the breeding colony and forage in areas with greater windspeeds and swell heights that correspond to seasonal mid-latitude westerlies in the North Pacific (Figs. S9, S10). The species nesting on Bird Island forage within the band of elevated windspeeds and swell heights corresponding with year-round mid-latitude westerlies in the Southern Ocean. In both study areas, the foraging areas of albatrosses are constrained to areas with fast winds and high waves despite having access to areas with milder conditions.

### Wind and wave conditions of foraging areas used during the breeding season

**Fig. 5:**
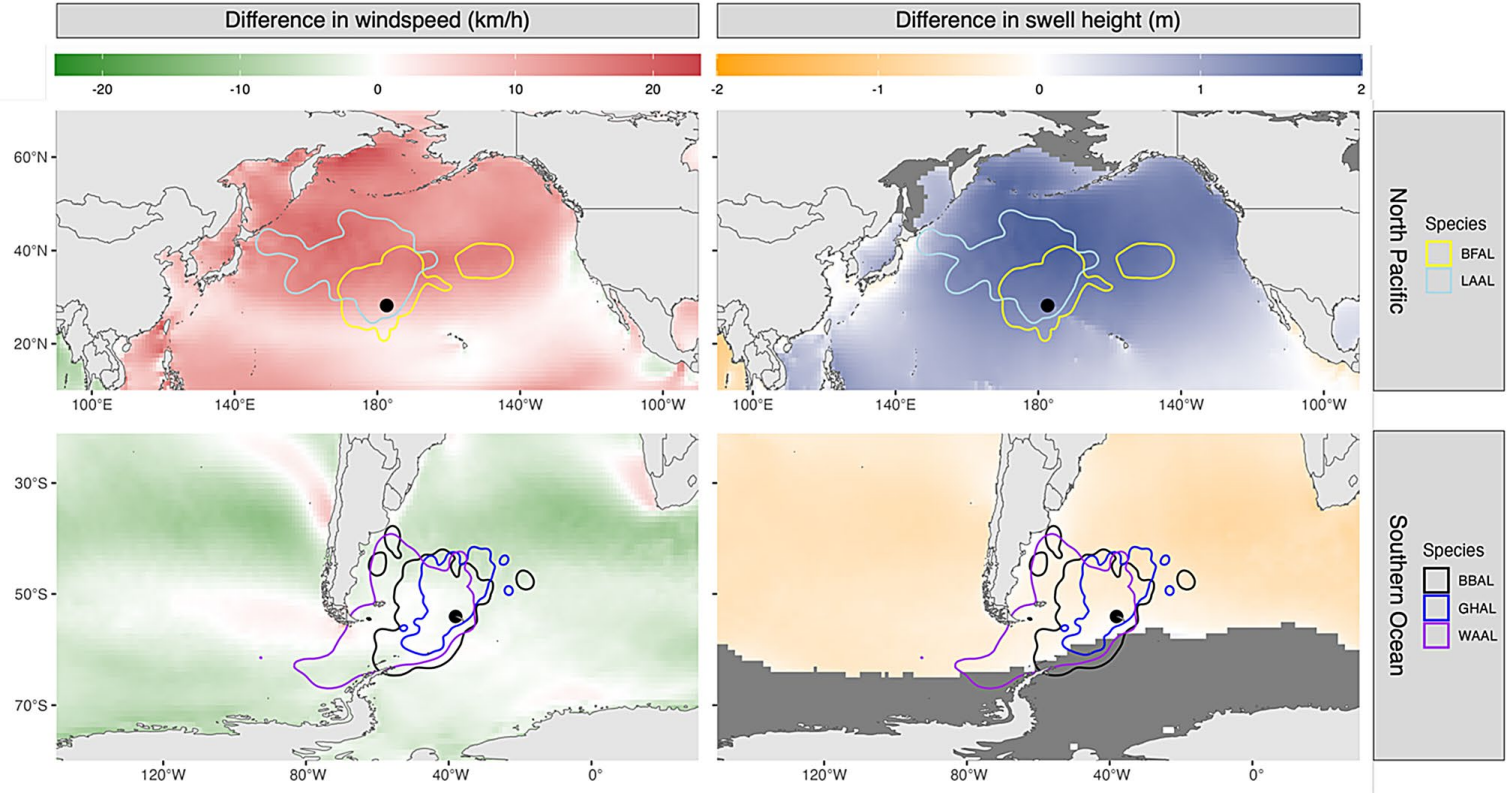
The difference in average windspeed and swell height between months in the breeding season (December, January, and February) and non-breeding months (here shown for June, July, August) of the years of the study (2018–2023). Positive values reflect higher windspeeds and swell heights during the breeding season. Lines represent the 95th percentile KDEs of foraging tracks and black circles represent the colony locations. Dark grey areas indicate the absence of data at that location

**Fig. 6:**
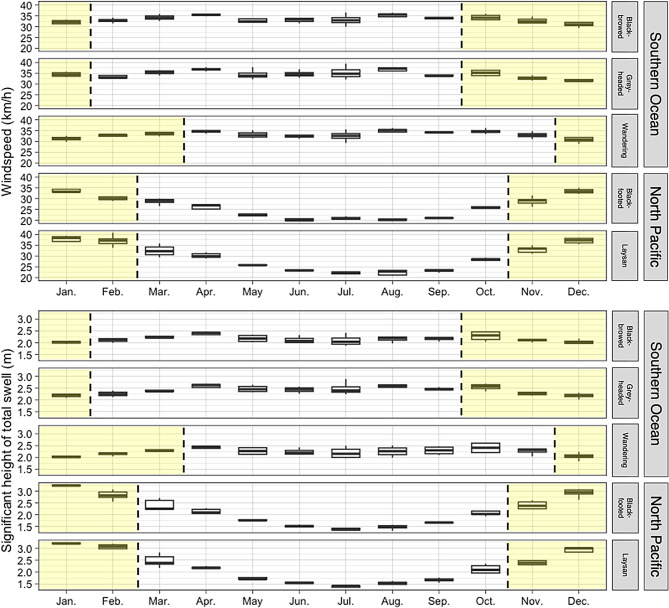
Seasonal changes to windspeed and swell height in a fixed area representing the foraging areas of each species used during the breeding season (i.e., the 95% KDEs). The yellow shading highlights months corresponding to the incubation and brood-guard periods for a given species

Using the breeding season foraging areas (KDEs) to identify local changes in wind and wave conditions through the year revealed a much greater seasonal variability within the KDEs of the North Pacific relative to those of the Southern Ocean. The greatest windspeeds and swell heights in the breeding season foraging areas of the North Pacific occurred between November and February during the incubation and brood-guard stages (Figs. 5, 6). In contrast, windspeed and swell height were more consistent throughout the year in the foraging areas used by the Southern Ocean species during the breeding season.

## Discussion

Flapping is an energetically expensive behavior that albatrosses avoid by soaring, allowing them to incur some of the lowest flight costs of any seabird while covering great distances over the open ocean [8, 22]. Despite their affinity for efficient soaring flight, albatrosses still use flapping flight when wind conditions are not sufficient for efficient or sustained soaring [17, 19] and also interject occasional flaps into soaring cycles to assist stability and lift [24]. Our study is the first to take advantage of advances in biologging to capture changes in albatross flight behavior relative to both wind and wave conditions and compare these changes across species and across ocean basins. We analyzed more than 38,000 hours of sensor data from 370 foraging albatrosses to determine how five species in two different ocean basins can reduce their energetic costs by limiting flapping flight via the use of wind and waves to soar. Our estimates show that soaring using wind and waves in tandem can reduce flap rates by 89–93% (representing a reduction of 974–1792 flaps per hour), suggesting considerable reductions in energy expenditure due to reduced flapping.

Theoretical studies have highlighted that albatrosses need sufficient winds for dynamic soaring [18, 19]. In the Southern Ocean, our analyses confirmed declines in flapping rates as windspeeds increased, likely indicating an increase in soaring relative to flapping flight, similar to that observed by Schoombie et al. [24] for wandering albatrosses. Declines in flapping rates with increasing windspeed were similar across Southern Ocean species, despite considerable variability in body size and wing morphology. However, relationships between flapping rates and windspeeds were less clear for black-footed and Laysan albatrosses, indicating that winds alone do not explain their flight behavior. Instead, swell height was a better predictor of flight behavior for North Pacific species, where flap rates declined with increasing swell height. Together these results suggest that North Pacific albatrosses rely more heavily on wave-slope soaring than dynamic soaring, whereas Southern Ocean albatrosses rely primarily on dynamic soaring.

Swell heights experienced by albatrosses in the North Pacific albatrosses were greater than those experienced by those in the Southern Ocean, whereas windspeeds were similar for all five study species. Richardson [19] estimated that the energy available from winds in typical Southern Ocean conditions is 4–5 times greater than that made available by waves. This aligns with our findings highlighting the use of wind-fueled soaring among Southern Ocean albatrosses, which primarily foraged in regions with relatively low swell heights ( < 2.2 m). However, the balance of energy available from wind versus waves may be different in the North Pacific. Black-footed and Laysan albatrosses spent most of their time foraging in regions of high swell heights (≥3.1 m), and swell height was a better predictor than windspeed of flap rate. Our results suggest that in the Southern Ocean albatrosses rely more heavily on winds to fuel dynamic soaring, likely because winds in this region provide more energy for soaring than waves. In contrast, North Pacific species appear to rely more heavily on waves to wave-slope soar, likely because higher swells in the North Pacific make waves a better source of energy than winds. Further analyses could estimate the differences in energy available from wave-slope versus dynamic soaring in the North Pacific given typical wind and wave conditions.

The Southern Ocean is typically considered to have the windiest and roughest wave field across global oceans (e.g. [69]) so our findings that North Pacific albatross experienced greater swell heights and similar windspeeds to Southern Ocean albatross were surprising. However, further analyses revealed that this occurred because Laysan and black-footed albatrosses breed in months when swell heights in their foraging habitat was maximal (Figs. 5, 6). While mean annual swell height and windspeed in proximity to Midway Atoll in the North Pacific are lower than those near Bird Island in the Southern Ocean (Fig. 1), there was considerable variability in windspeed and swell height in habitats used by breeding albatross in the North Pacific. In contrast, swell heights in habitats used by breeding Southern Ocean albatross were consistently high throughout the year (Figs. 5, 6). When Laysan and black-footed albatrosses are breeding, strong westerly winds extend further south in the North Pacific [1, 4], resulting in high windspeeds in lower-latitude habitats used by breeding albatross. During the non-breeding months, the North Pacific species typically use higher latitudes [62, 70], where the highest windspeeds in these time periods are located. Together, our findings highlight the greater importance of seasonal climate variation for North Pacific albatrosses. Efficient wind and wave-driven movement, aided by heightened windspeeds and swell heights, may play a role in successful reproduction when birds are constrained to return to their breeding sites regularly to relieve their mate during incubation or feed young chicks.

Wind is often considered to be the key factor which facilitates the low cost of travel for albatrosses, and our analysis confirms this with tagging data, but also highlights the importance of waves and the need to consider both wind and waves in studies of albatross energetics. Flap rate decreased with both increasing windspeeds and swell heights for all five study species. Models incorporating the relative angle of wind or swells performed better than competing models that only assessed the impact of windspeed or swell height, highlighting the importance of considering relative angle as soaring depends on the direction of winds or waves. However, model performance increased considerably when both wind and wave variables were included in the same model; these models were consistently the best performing models for all five albatross species. Together, our results demonstrate that while specific behavioral responses to wind and waves varied across species and ocean basins, all study species are using both wind and waves to soar and minimize flapping flight. This is especially true for the Southern Ocean study species which all show a near-symmetrical response along the windspeed and swell height axes.

Although our sample sizes to assess flapping responses were large for four of our study species, those for black-footed albatrosses were limited to 18 individuals. The results of our down-sampling analysis suggested that this sample size was unlikely to affect our conclusions. However, we did observe some variability in flapping responses relative to wind when down-sampling the grey-headed albatross data, which emphasizes the importance of considering sample size when assessing flight behavior in seabirds in general.

Wind is increasingly recognized as a major driver of seabird movement and foraging energetics [4, 12]. The present study demonstrates an approach that can be used to assess a proxy of energy expenditure across soaring seabird species to better understand the impacts of wind. Further, this work shows that both wind and waves must be considered to effectively understand locomotory strategies of albatrosses in response to their environment. This knowledge, given a rapidly changing global environment, may be beneficial in predicting impacts of wind and wave conditions on albatross populations. Changes to global wind patterns have been observed in recent decades and are forecasted to amplify in the future [12, 71–73]. A more comprehensive understanding of how wind and waves mediate the cost of travel for soaring seabirds is needed to more accurately predict how seabirds will respond to projected scenarios.

## Conclusions

Our findings suggest that both wind and waves play critical roles in shaping the energetics of albatross flight. By analyzing more than 38,000 hours of high-resolution sensor data across five albatross species in two ocean basins, we found that soaring using both wind and waves reduced wing flapping by 89–93%, suggesting substantial energetic savings. Regional differences in model performance of flap rates relative to environmental conditions suggests that Southern Ocean albatrosses may primarily rely on wind-driven dynamic soaring, where North Pacific albatrosses are more reliant on wave-slope soaring. These behavioral differences across ocean basins reflect how species adapt their movement strategies to local environmental conditions and underscore the need to consider both wind and wave dynamics when evaluating seabird foraging energetics. Our findings emphasize that the physical environment governs the locomotory costs of seabirds, highlighting the need for a deeper understanding of the impact of wind and waves on efficient flight to anticipate the vulnerability and resilience of albatross populations.

## Electronic supplementary material

Below is the link to the electronic supplementary material.

Supplementary Material 1

## Data Availability

Hourly summaries of flap rates and wind and wave conditions used to produce models are available in Dryad (10.5061/dryad.v15dv429p)

## Abbreviations

ERA5: European Centre for Medium-Range Weather Forecasts Reanalysis v5
GAM: Generalized Additive Model
GLS: Global Location Sensor
GPS: Global Positioning System
HMM: Hidden Markov Model
KDE: Kernel Density Estimate
