# Supplementary material for: Differential impacts of wind and waves on albatross flight performance in two ocean basins
Source: Mov Ecol. 2025 Dec 20;14:1. doi: 10.1186/s40462-025-00614-w (PMC12781567; doi:10.1186/s40462-025-00614-w)
Supplement: Supplementary file 1 — Supplementary Material 1 [file 40462_2025_614_MOESM1_ESM.docx]

**Supplemental Information for:**

Differential impacts of wind and waves on albatross flight performance in two ocean basins

**Materials and Methods (Supplemental Information)**

*Characterizing flapping behavior*

To count individual flaps, we followed Schoombie’s (2023) framework of detecting flaps in the z-axis of accelerometer data, by first cleaning the raw heave signal using LULU operators—see Lotz and Clilverd (2019)—to filter out pulses that were greater than *m* samples in width, and then detecting peaks above a threshold (*th*) to count flaps (**Figure s1**). To select *m* and *th*, *m* was initially set to 8 samples (~3 Hz), which is the flapping frequency of wandering albatross (Sato et al., 2009) and *th* was incremented by increments of 0.05 from -1.0 to -0.3 g, for which the total number of flaps was recorded for each value of *th* (**Figure s1**). Plotting the number of flaps as a response to *th*, the moving slope was calculated for each value of *th*. The *th* corresponding to the smallest moving slope, or the *th* at which the number of flaps changed the least, was chosen as the threshold to detect flaps. This was set as close to zero as possible without detecting noise from the z-axis signal. Next, *th* was held at the chosen value, and *m* was increased from 7 to 11 by increments of 1 to select the *m* that led to the smallest moving slope of the number of flaps. This ensures that *m* was high enough to let flap signals through the filter but not too high such that noise is also being let through.

*Modeling relationships between flap rates and wind and wave parameters*

All models used three knots for wind and wave variable smooths to avoid overfitting, used REML estimation and a log link function to fit a negative binomial distribution, and included individual as a random effect smooth with the number of knots equal to the number of individuals. A negative binomial distribution was chosen given the high variance relative to the mean of the flap rate data, which were discrete values.

For Model I and II, we used a thin plate regression spline for windspeed and swell height, respectively. For Model III we fit a full tensor product smooth (“te” in the *mgcv* package) of windspeed and bird-wind angle as a continuous variable (0-180º) and used the same approach for Model IV (full tensor product smooth of swell height and bird-swell angle as a continuous variable). For model V, we used a full tensor product smooth of windspeed and swell height. A full tensor product includes marginal effects and interactions of the inputted variables and are comprised of tensor product interactions (“ti” in the *mgcv* package) such that the full tensor product of variables a and b can be broken down into three tensor product interactions: ti(a) + ti(b) +ti(a,b). We selected this approach for model III and IV to account for independent effects of wind/wave magnitude and relative angle on flap rate, as well as the effects of the interaction between these variables, as flap rate relative to wind or wave magnitude may vary with relative angle. Similarly, for Model V, using the full tensor product smooth accounted for effects of windspeed and swell height on flap rate, as well as the interaction between these two variables. Model V did not assess all possible interactions between windspeed, bird-wind angle, swell height, and bird-swell angle because a model with this level of complexity would be difficult to interpret. Further, models including bird-wind angle or bird-swell angle only showed incremental improvements in R^2^ and deviance explained from models including only windspeed and swell height (see Results section in main text), highlighting that windspeed and swell height were the most important variables to model together.

**Results (Supplemental Information)**

*Flap rate models – Models I–IV*

Model I, which uses windspeed to predict flap rate, revealed that all three Southern Ocean albatross species showed a similar response in flapping rates relative to winds, where flap rate decreased as windspeed increased (**Figure s4**). In contrast to the Southern Ocean species, the two North Pacific species did not show consistent declines in flap rates with increasing windspeed, and the responses to wind differed between the two North Pacific species. Model II, which uses swell height to predict flap rate, showed that flap rates for black-browed and grey-headed albatrosses plateaued as swell heights rose from low to intermediate, and then decreased as swell heights rose from intermediate to high (**Figure s4**). The flap rate of wandering albatrosses increased to a peak at intermediate swell heights before decreasing to a minimum for the maximum swell height. Both North Pacific species showed flap rates that declined with increasing swell height. Models III and IV, which use both the magnitude and relative angle of wind and swell to predict flap rate, demonstrate that for most albatrosses, magnitude typically had a larger effect on flapping rate than relative angle (**Figure s5**). Across different windspeeds, albatrosses generally showed increased flap rates in head winds (**Figure s5**). For grey-headed and wandering albatrosses, flap rates increased in sidewinds at the lowest windspeeds, while for Laysan albatrosses, flap rates increased in sidewinds at the highest windspeeds (**Figure s5**). Across different swell heights, albatrosses generally showed somewhat higher flap rates when oriented against the waves (**Figure s5**).

**References (Supplemental Information)**

Lotz, S. I., & Clilverd, M. (2019). Demonstrating the Use of a Class of Min‐Max Smoothers for *D* Region Event Detection in Narrow Band VLF Phase. *Radio Science*, *54*(3), 233–244. https://doi.org/10.1029/2018RS006701

Sato, K., Sakamoto, K. Q., Watanuki, Y., Takahashi, A., Katsumata, N., Bost, C.-A., & Weimerskirch, H. (2009). Scaling of Soaring Seabirds and Implications for Flight Abilities of Giant Pterosaurs. *PLoS ONE*, *4*(4), e5400. https://doi.org/10.1371/journal.pone.0005400

Schoombie, S., Wilson, R., & Ryan, P. (2023). Wind driven effects on the fine-scale flight behaviour of dynamic soaring wandering albatrosses. *Marine Ecology Progress Series*, *723*, 119–134. https://doi.org/10.3354/meps14265

**Tables (Supplemental Information)**

**Table s1** Terms used in GAMs.

| Model | Environmental term | Smoothing term used |
| --- | --- | --- |
| 0 | n/a | n/a |
| I | Windspeed | Thin plate regression spline |
| II | Swell height | Thin plate regression spline |
| III | Windspeed, BWA | Full tensor product smooth |
| IV | Swell height, BSA | Full tensor product smooth |
| V | Windspeed, Swell height | Full tensor product smooth |

The GAMs predict flap rate using a random effect smooth for individual variation, where the number of knots is equal to the number of individuals, and the environmental term given in the table, which either has one or two variables.

**Table s2** Tag Deployments

| **Species** | **Trip Type** | **Mean trip duration (days)** | **Mean acc duration (days)** |
| --- | --- | --- | --- |
| Black-browed albatross | Brood-guard | 3.6 | 3.6 |
|  | Incubation | 11.3 | 10.1 |
| Grey-headed albatross | Brood-guard | 3.8 | 3.6 |
|  | Incubation | 10.5 | 9.8 |
| Wandering albatross | Brood-guard | 3.5 | 3.4 |
|  | Incubation | 13.7 | 9.5 |
| Black-footed albatross | Brood-guard | 2.6 | 2.5 |
|  | Incubation | 13.9 | 7.9 |
| Laysan albatross | Brood-guard | 3.5 | 3.2 |
|  | Incubation | 13.4 | 11.8 |
| **All** | Brood-guard | 3.6 | 3.5 |
|  | Incubation | 11.8 | 10.1 |

Mean duration of GPS and accelerometer deployments trip durations during brood-guard and incubation breeding stages. Durations start when the bird leaves the colony and end when the tag stops recording or when they return to the colony (whichever is first).

**Figures (Supplemental Information)**


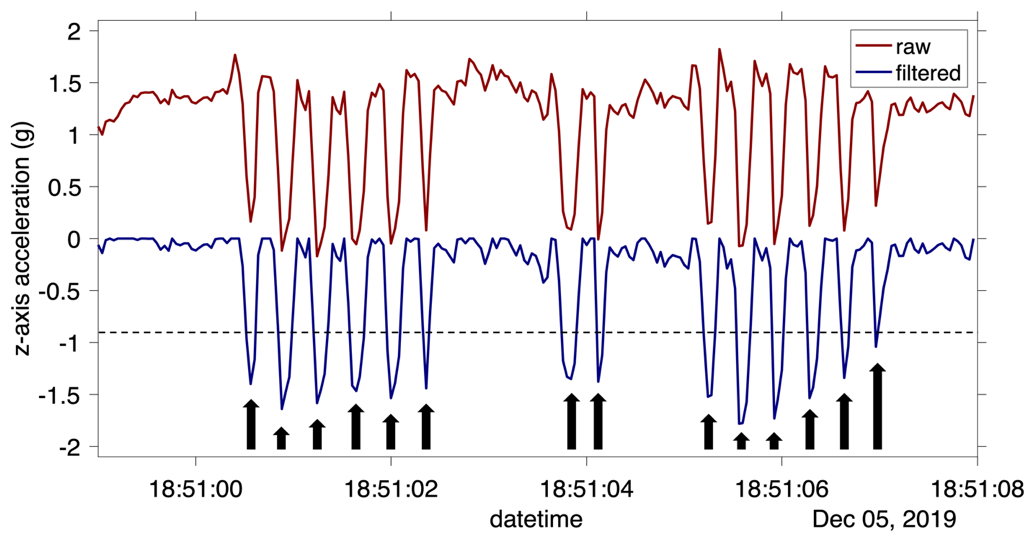


**Figure s1** Flap detection methodology. Top panel: filtering z-axis acceleration using a LULU filter. In this instance, the LULU filter used an upper limit (m) of 9. As indicated by the dotted line, the threshold (th) applied to detect flaps (black arrows) is -0.9. Bottom panel: the total number of flaps detected while holding m at 8 and incrementing th from -1.0 to -0.3 by intervals of 0.05. The threshold (th) is selected as the value at which the moving slope of total flap count is smallest: -0.9 in this example.

*
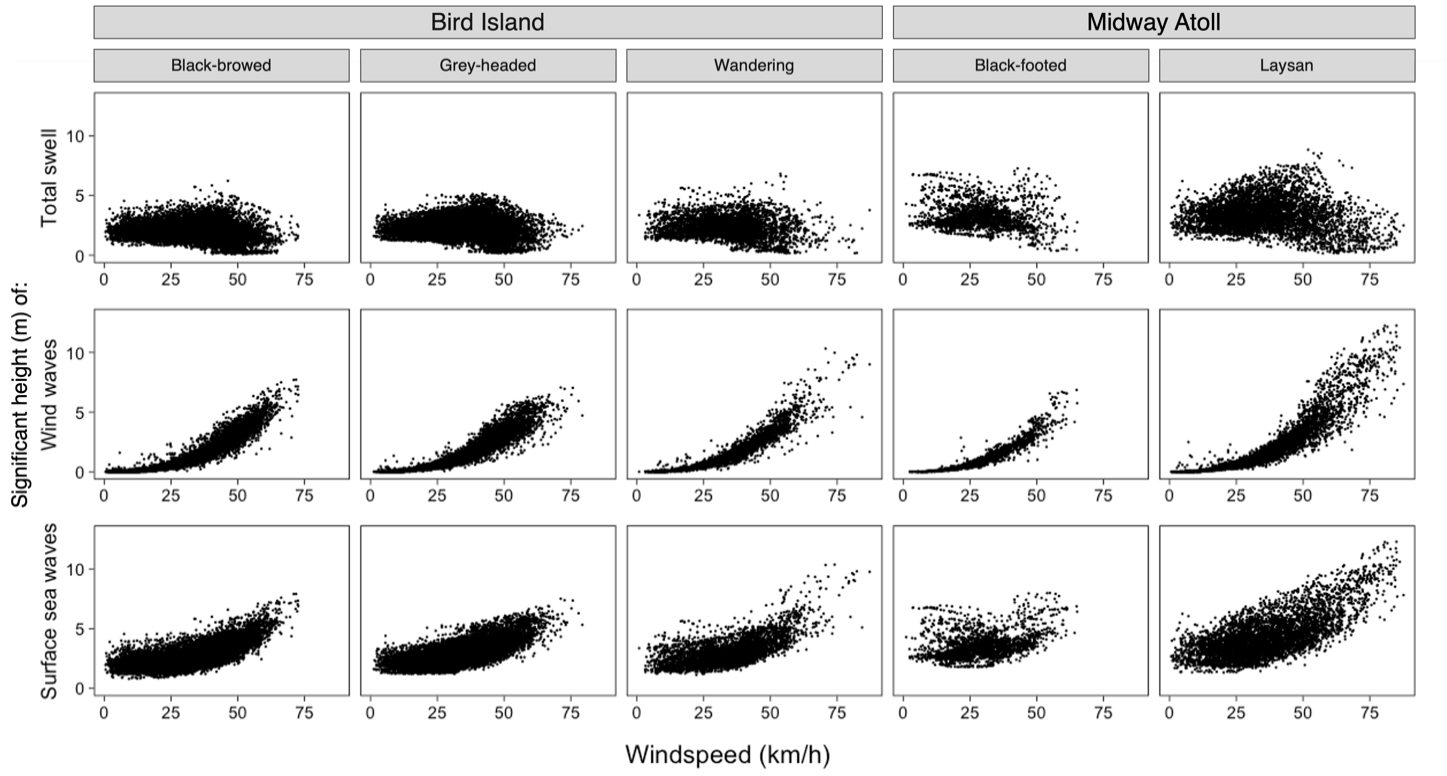
*

**Figure s2** The relationship between windspeed and the significant height of waves. Total swell (top row) is uncorrelated to windspeed, while wind waves are directly influenced by windspeeds.

*
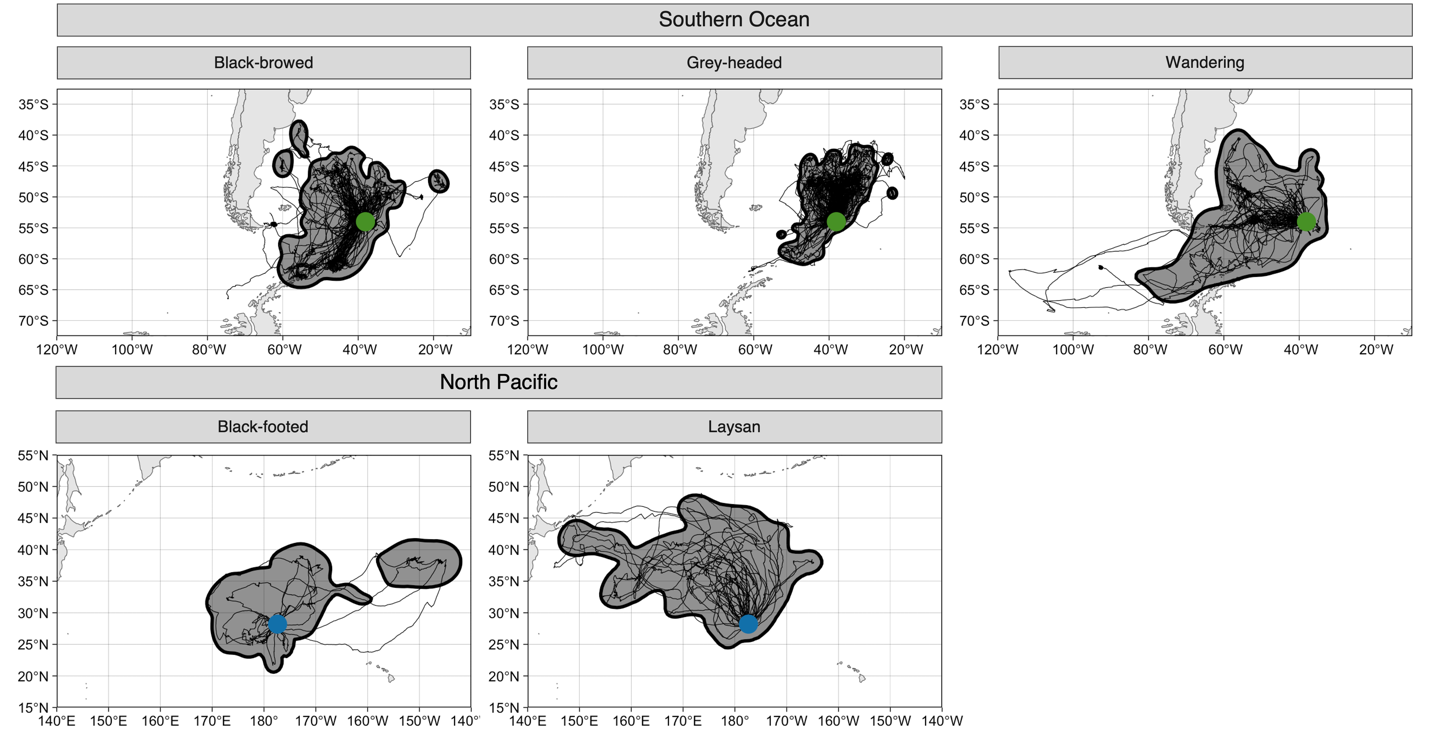
*

**Figure s3** General foraging areas during nesting created using 95th percentile KDEs of foraging tracks. Thin lines represent foraging tracks, bold lines and the enclosed shaded areas represent the KDEs, and dots represent colonies (green = Bird Island, blue = Midway). KDEs were used to assess changes in wind during the year.


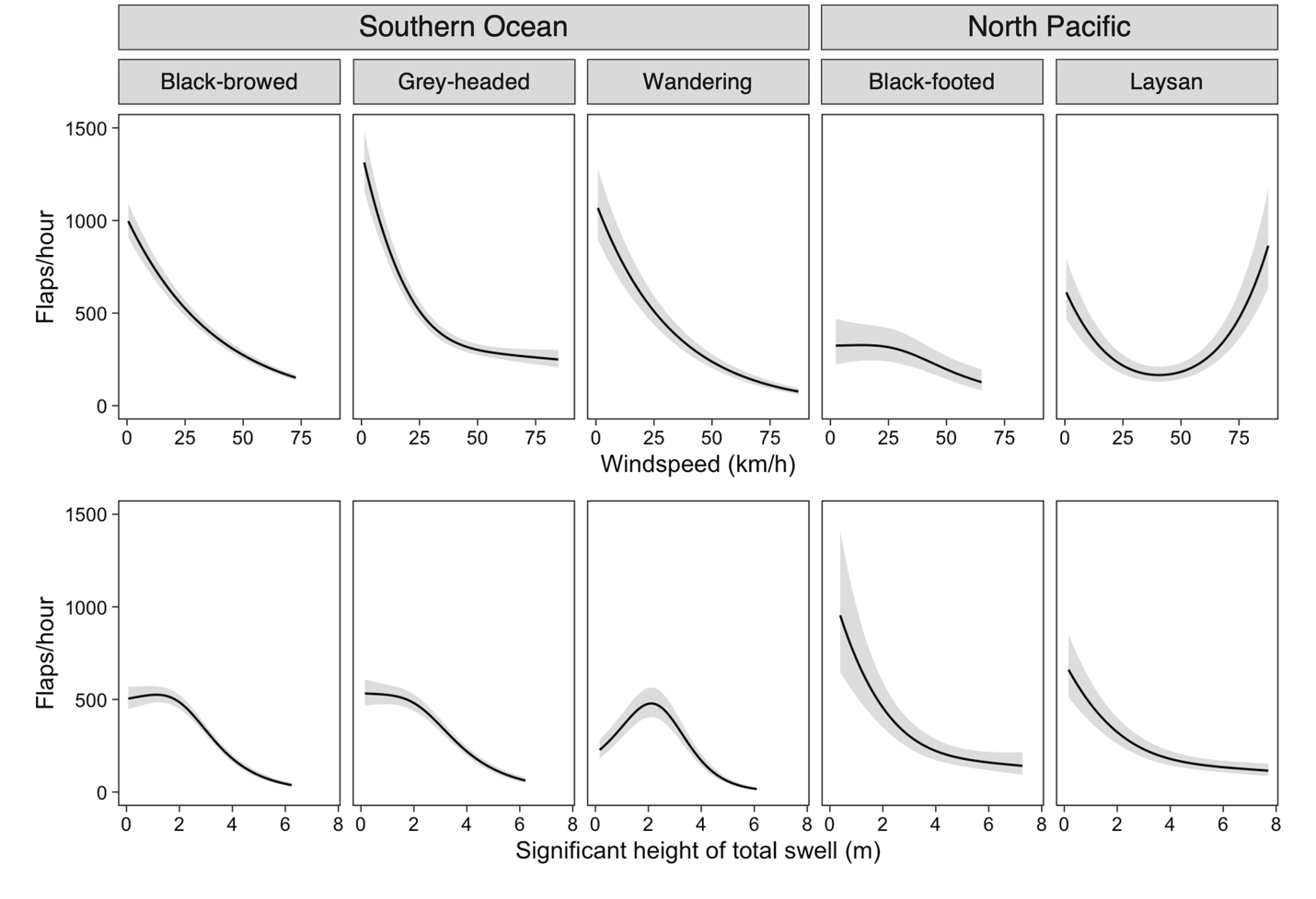


**Figure s4** GAMs using thin plate regression splines to predict flap rate using the magnitude of wind or swell. All figures display the GAM predictions and 95% confidence intervals. The top row displays the outputs of Model I, which uses windspeed alone. The bottom row displays the outputs of Model II, which uses swell height alone.


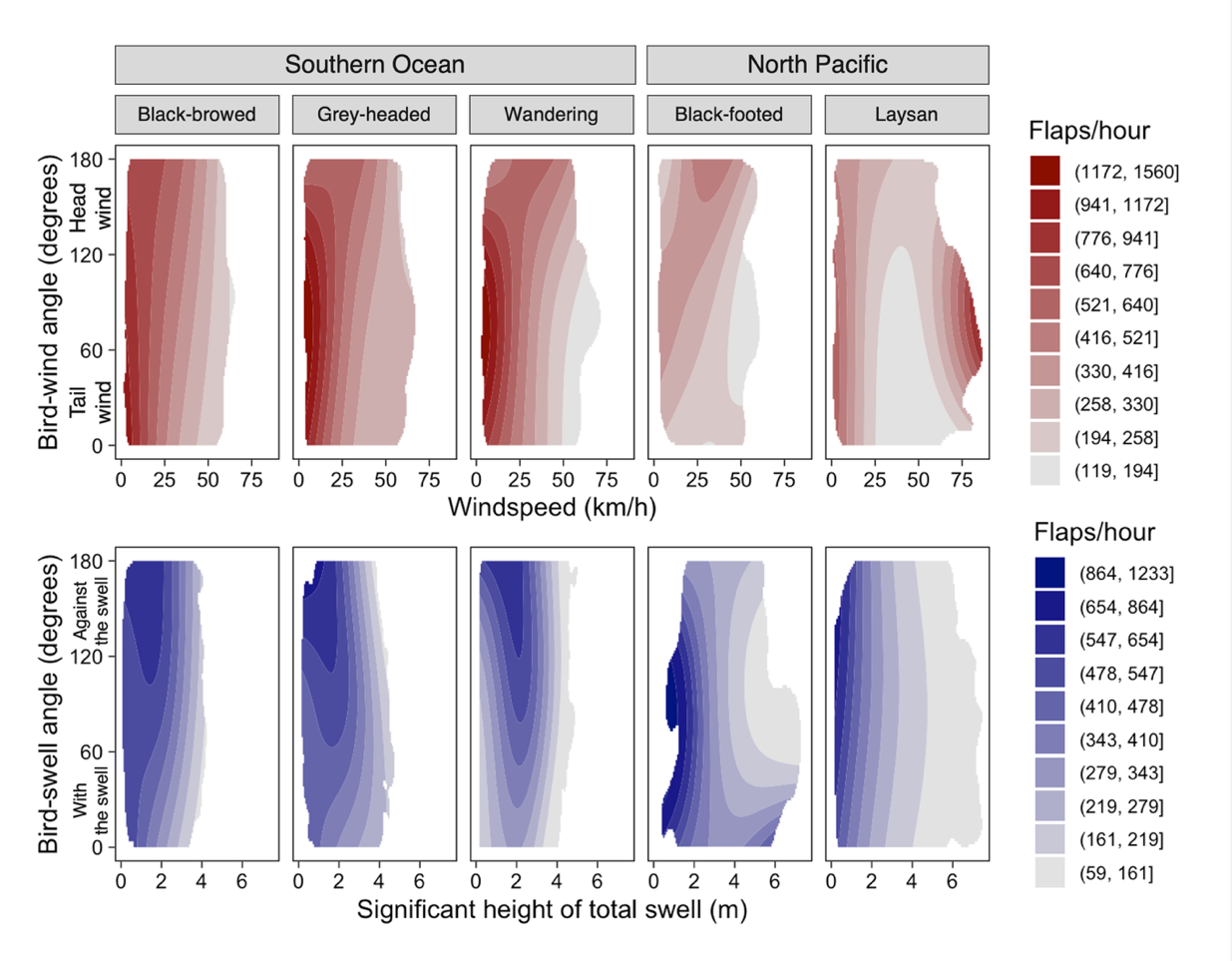


**Figure s5** GAMs predicting flap rate using the magnitude and relative angle of wind or swell (Models III and IV). Figures display the GAM predictions using colored contours created using Jenks’ natural breaks. The binned GAM responses that lack a corresponding color indicate that no model outputs across the predictor space fell into these bins. The top row (red) displays the outputs of Model III, which uses windspeed and bird-wind angle. The bottom row displays the outputs of Model IV, which uses swell height and bird-swell angle. GAM outputs were confined to the 99% kernel density estimate of the predictor variable space to avoid interpreting the output of environmental conditions that are impossible or unlikely. For most albatrosses, magnitude had a larger effect on flapping rate than relative angle, seen in the contour plots as greater change in flap rate along the x axis than the y axis.

*
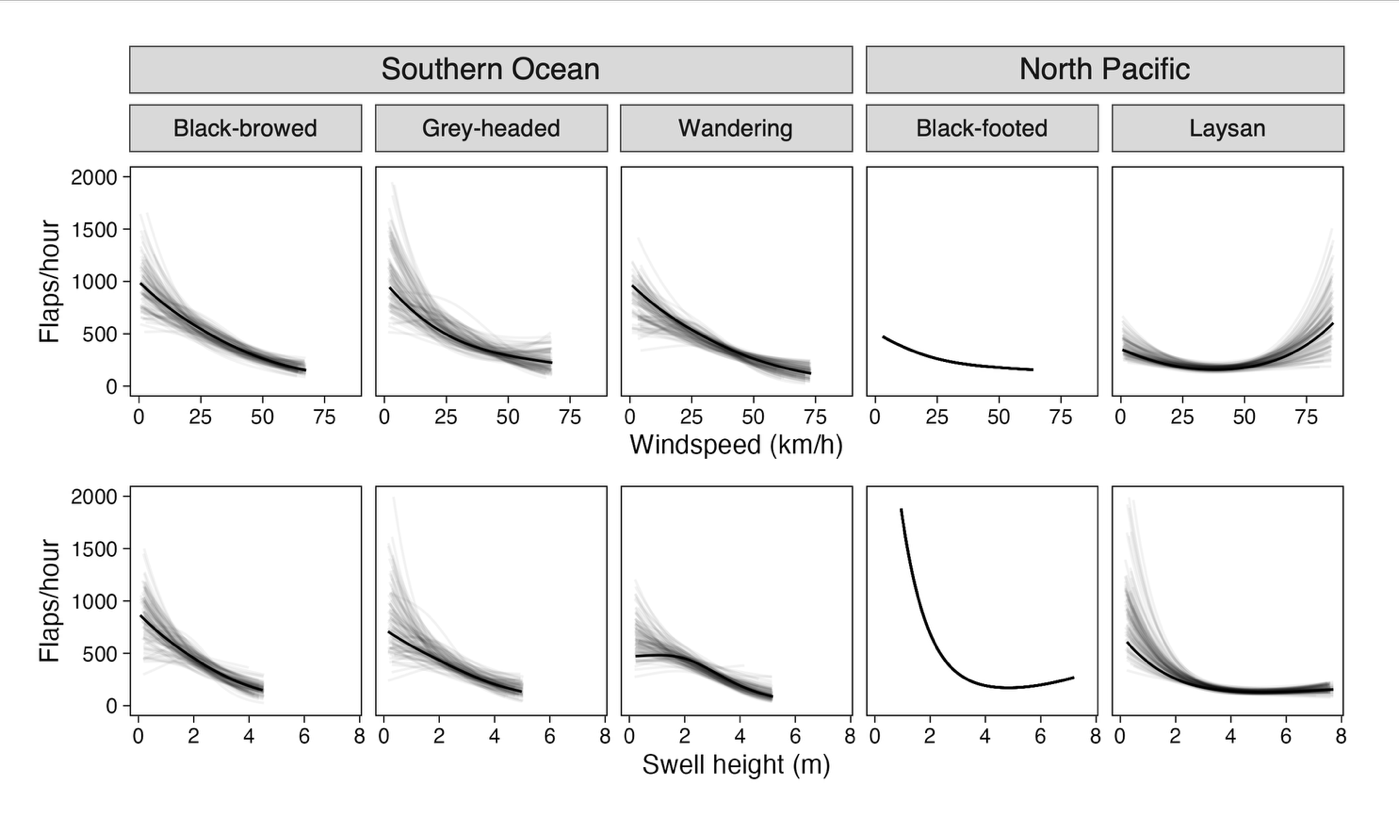
*

**Figure s6** GAMs predicting flap rate using down-sampled datasets. GAM predictions were created using 100 iterations of a randomly selected down-sampled dataset of 18 individuals from each species, reflective of the sample size available for black-footed albatross. All figures display GAM prediction curves created using the full dataset in bold along with the 100 down-sampled prediction curves in a lighter shade. The top row displays Model V predictions with swell height held constant at the mean experienced value while the bottom row displays Model V predictions with windspeed held constant at the mean experienced value.

*
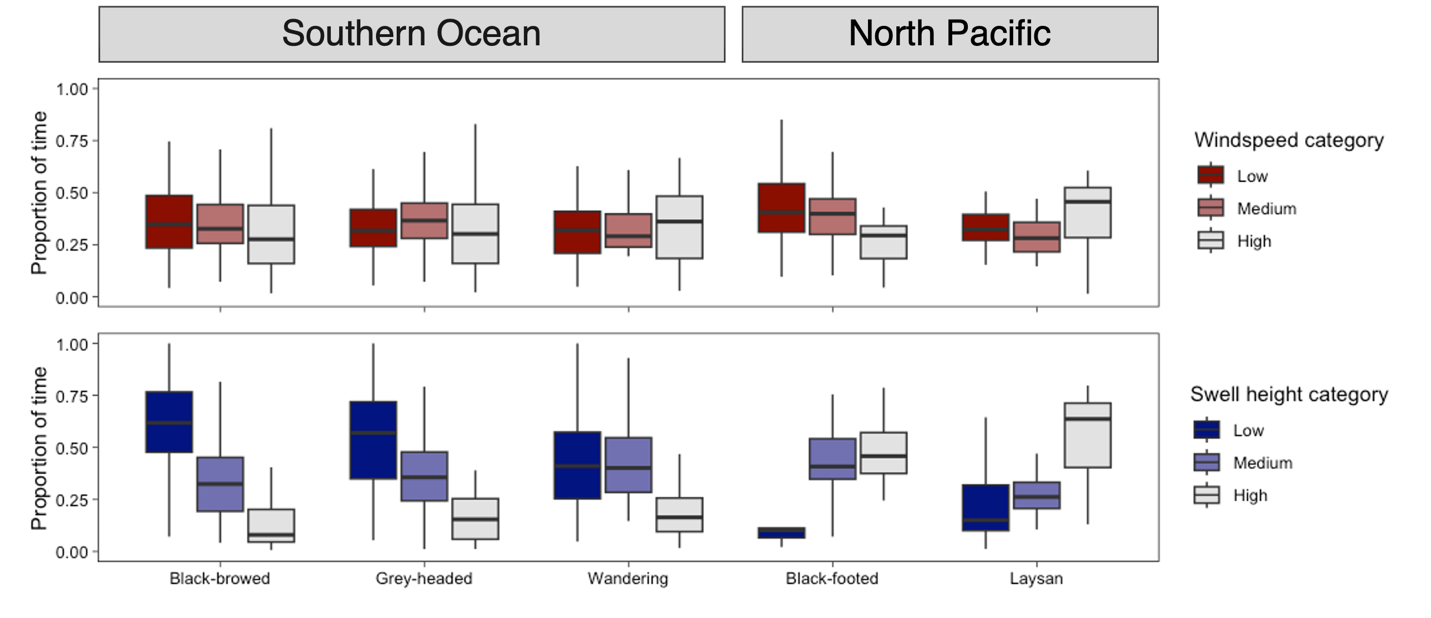
*

**Figure s7** Boxplots representing the proportion of time individuals from each species spend in low, medium, high windspeeds and swell heights, respectively.

*
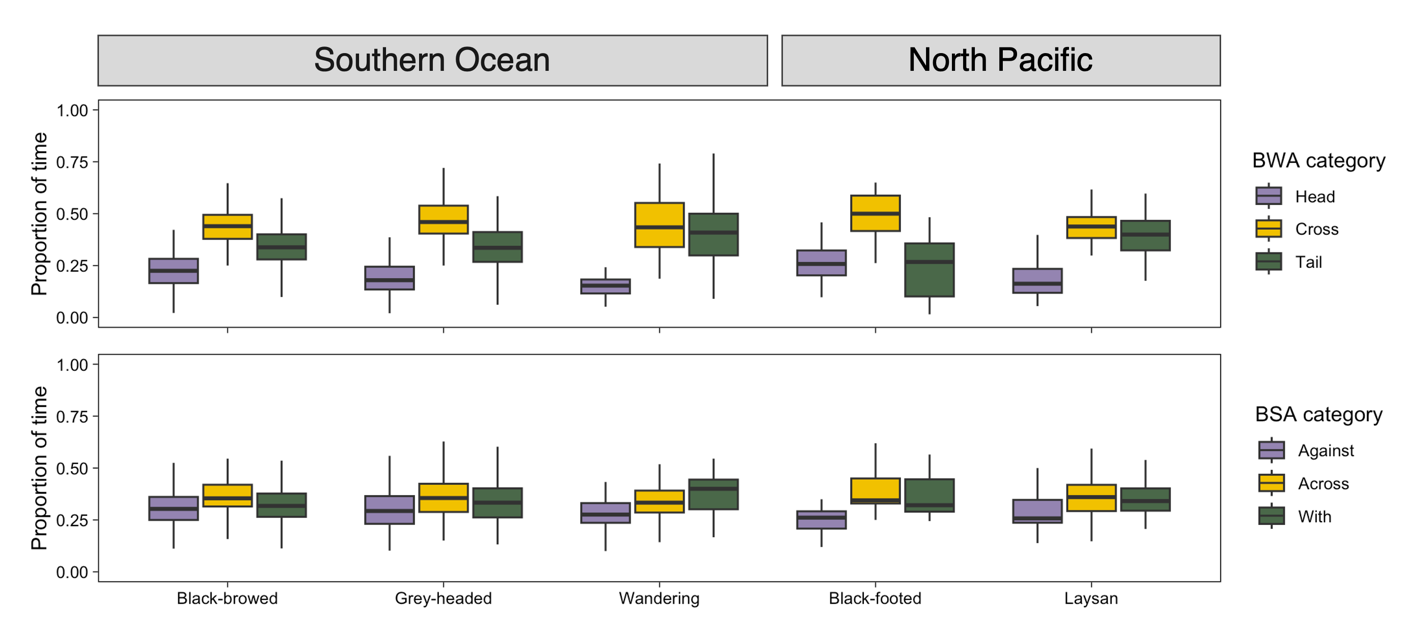
*

**Figure s8** Boxplots representing the proportion of time individuals from each species spend in relative wind and wave angles. BWA (bird-wind angles) are classified as tailwinds (<60º), crosswinds (≥60º & <120º), and headwinds (≥120º), while BSA (bird-swell angles) are classified as with (<60º), across (≥60º & <120º), and against (≥120º) the wind.

*
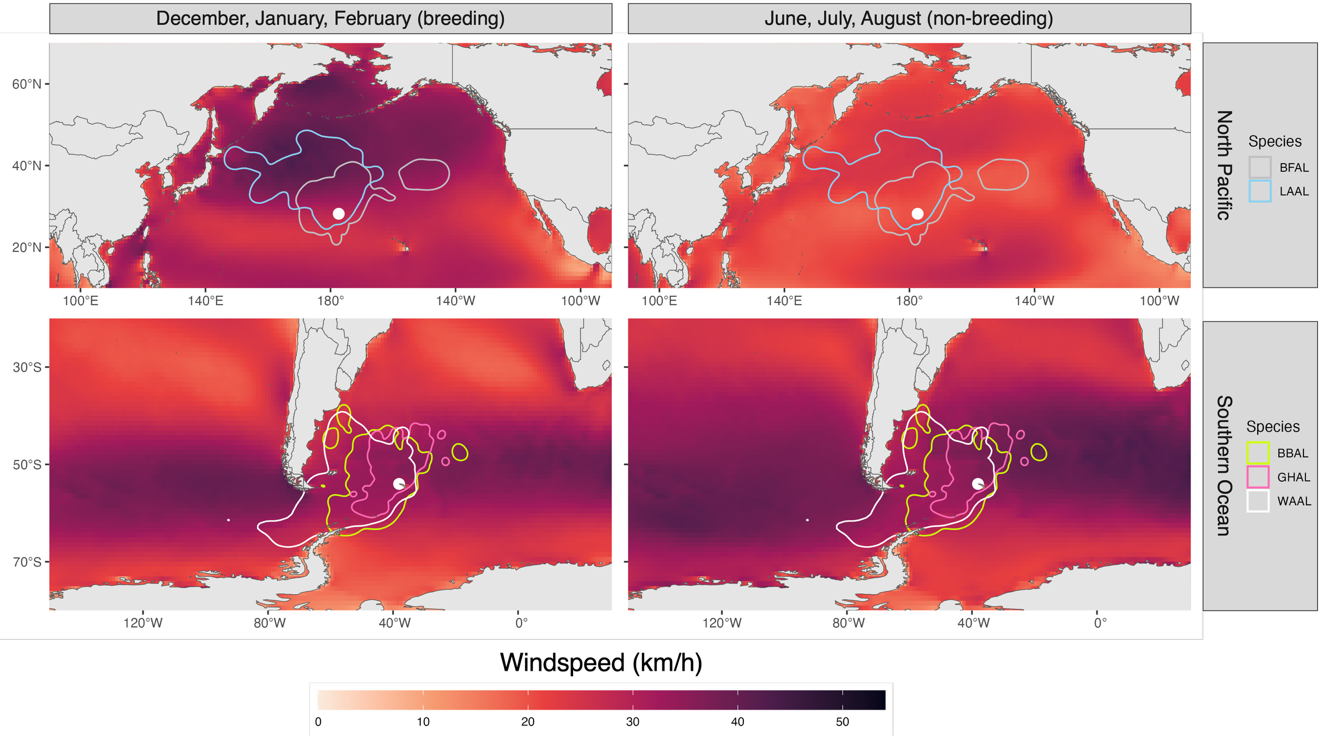
*

**Figure s9** Average windspeed during breeding season months (December, January, and February) and non-breeding months (here shown for June, July, August) of the years of the study (2018-2023). Lines represent the 95^th^ percentile KDEs of foraging tracks and white circles represent the colony locations.

*
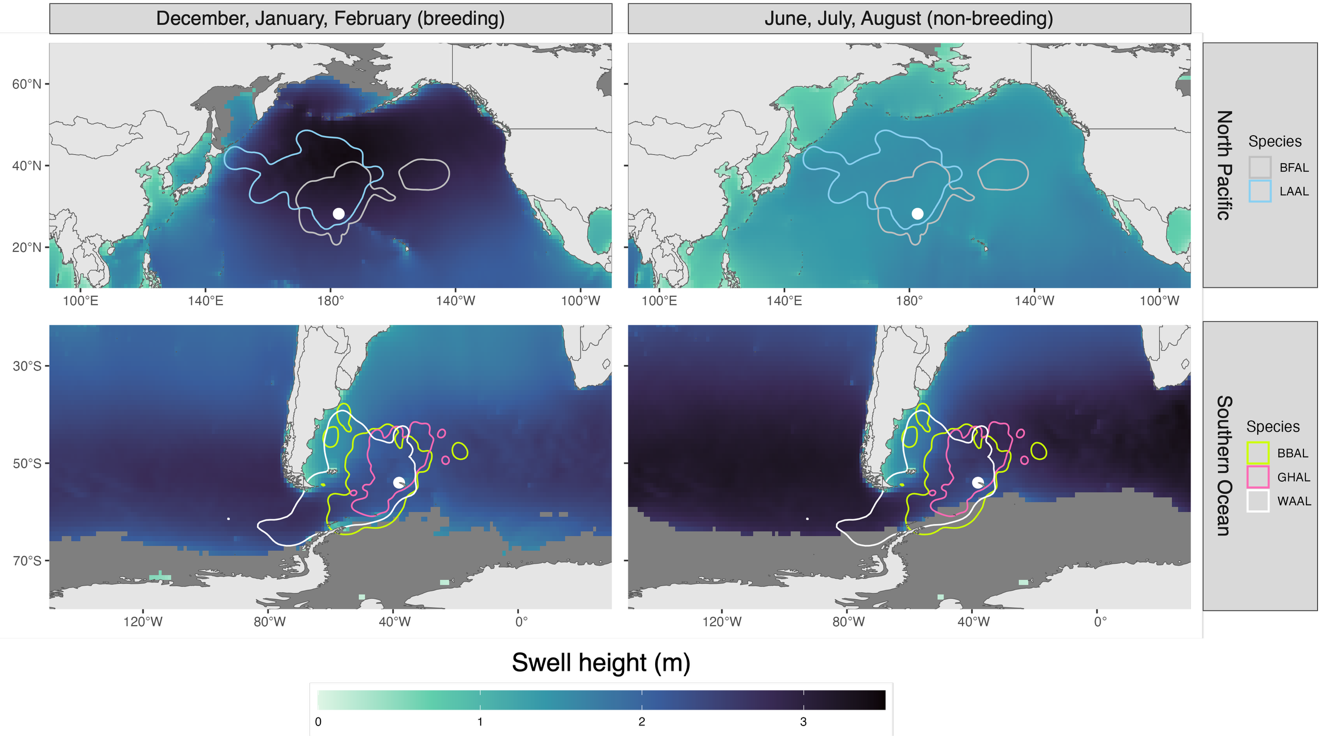
*

**Figure s10** Average swell height during breeding season months (December, January, and February) and non-breeding months (here shown for June, July, August) of the years of the study (2018-2023). Lines represent the 95^th^ percentile KDEs of foraging tracks and white circles represent the colony locations. Dark grey areas indicate a lack of data at that location.
